# Microscopic and spectroscopic bioassociation study of uranium(VI) with an archaeal *Halobacterium* isolate

**DOI:** 10.1371/journal.pone.0262275

**Published:** 2022-01-13

**Authors:** Stephan Hilpmann, Miriam Bader, Robin Steudtner, Katharina Müller, Thorsten Stumpf, Andrea Cherkouk

**Affiliations:** Institute of Resource Ecology, Helmholtz-Zentrum Dresden-Rossendorf, Dresden, Germany; Central Agricultural University, INDIA

## Abstract

The safe disposal of high-level radioactive waste in a deep geological repository is a huge social and technical challenge. So far, one of the less considered factors needed for a long-term risk assessment, is the impact of microorganisms occurring in the different host rocks. Even under the harsh conditions of salt formations different bacterial and archaeal species were found, *e*. *g*. *Halobacterium* sp. GP5 1–1, which has been isolated from a German rock salt sample. The interactions of this archaeon with uranium(VI), one of the radionuclides of major concern for the long-term storage of high-level radioactive waste, were investigated. Different spectroscopic techniques, as well as microscopy, were used to examine the occurring mechanisms on a molecular level leading to a more profound process understanding. Batch experiments with different uranium(VI) concentrations showed that the interaction is not only a simple, but a more complex combination of different processes. With the help of *in situ* attenuated total reflection Fourier-transform infrared spectroscopy the association of uranium(VI) onto carboxylate groups was verified. In addition, time-resolved laser-induced luminescence spectroscopy revealed the formation of phosphate and carboxylate species within the cell pellets as a function of the uranium(VI) concentration and incubation time. The association behavior differs from another very closely related halophilic archaeon, especially with regard to uranium(VI) concentrations. This clearly demonstrates the importance of studying the interactions of different, at first sight very similar, microorganisms with uranium(VI). This work provides new insights into the microbe-uranium(VI) interactions at highly saline conditions relevant to the long-term storage of radioactive waste in rock salt.

## Introduction

Since the 1950s, nuclear power plants have been used commercially for energy generation [1]. The final disposal of the resulting high-level radioactive waste represents one of the largest scientific and social challenges of our time. Different approaches are taken into consideration worldwide [2]. Rock salt is a possible host rock for the long-term storage of this waste, besides clay and crystalline rock [3]. In addition to the geological, geochemical and geophysical properties of rock salt it is also necessary to extend knowledge about the indigenous microorganisms and their influence on the final disposal of the radioactive waste [4]. Considering a worst-case scenario, water can penetrate the repository, especially after a longer period of storage. As a result, radionuclides can enter the surrounding host rock and interact with microorganisms. Microbes can influence the solubility and hence, the mobility of radionuclides by different processes, *e*. *g*. sorption, reduction/oxidation, accumulation or change of the speciation [5–8]. The combination of these processes can be described simplified as cell-related bioassociation.

It is well-known that microorganisms occur in different high saline environments such as rock salt or salt lakes [9–11]. They can survive long periods under these extreme conditions, which also includes a lack of nutrients [11–13]. In this work we investigated the interactions of an extremely halophilic archaeon, *Halobacterium* sp. GP5 1–1, with uranium(VI), which has been isolated from a German rock salt sample. The sequence of the 16S rRNA gene is shown in S1 Dataset. A comparison of this gene sequence with the sequences deposited in the BLAST database [14] showed a very close relation to *Halobacterium hubeiense* JI20-1, which was isolated by Jaakkola *et al*. from a 123 million-year-old rock salt from China [15]. Furthermore, *Halobacterium noricense* DSM15987^T^, which was also isolated from a rock salt sample (Altaussee, Austria), is another closely related strain based on the 16S rRNA gene sequence, varying only in one base pairing over 818 base pairs (bp) of the 16S rRNA gene [16]. A phylogenetic dendrogram including all the 16S rRNA gene sequences of the above mentioned archaea is shown in S1 Fig. The interactions of *Halobacterium noricense* DSM15987^T^ with uranium(VI) were already investigated in detail [17–19]. The aim of this study was to get more information about the interaction mechanisms of a closely related microorganism (*Halobacterium* sp. GP5 1–1) to this one. With the help of these investigations, we wanted to find out whether it is important to study the microbe-radionuclide interactions of more than one closely related species in order to achieve a comprehensive safety concept for a repository in the deep geological subsurface.

Uranium is the major component of high-level radioactive waste. Therefore, it is crucial to know which interaction mechanisms with microorganisms occur when this radionuclide is released. Moreover, the uranyl(VI) cation is isostructural to plutonyl(VI), hence it can act as an analogue. Plutonium plays an important role for the safety of the final disposal site due to its high radiotoxicity [20].

In this study, we investigated the bioassociation behavior of different uranium(VI) concentrations onto the cells of the halophilic archaeon *Halobacterium* sp. GP5 1–1 using a combined approach of one microscopic and various spectroscopic techniques. In this way, we aimed to understand the interaction mechanisms on a molecular level. Using *in situ* attenuated total reflection Fourier-transform infrared spectroscopy (ATR FT-IR) we can monitor the binding motives of uranium(VI) onto the cells within the first two hours of the bioassociation process. Time-resolved laser-induced fluorescence spectroscopy (TRLFS) shows the changes in the uranium(VI) speciation in supernatants and cell pellets over the whole incubation time. With fluorescence microscopy we carried out live/dead staining to get information about the viability of the cells during the experiment. Only the careful combination of the methods gives a new molecular insight into the ongoing interaction mechanisms and the formed uranium(VI) species. This information will contribute to a comprehensive safety assessment considering also geomicrobiology for the selection of a final disposal site for high-level radioactive waste in rock salt.

## Materials and methods

### Cultivation

A German rock salt sample was collected as described by Bader *et al*. [17]. A specific portion of the sample was incubated in three different sodium chloride concentrations (2 M, 3 M and 4 M) of modified R2A resuscitation buffer at room temperature. After an incubation time of 24 h, 300 μL of these buffers were spread on corresponding agar plates containing modified R2A medium with the respective sodium chloride concentrations and were incubated at 37 °C in the dark. Selected colonies were transferred to new plates and afterwards in MR2A liquid medium to get individual isolates as *Halobacterium* sp. GP5 1-1. It was furthermore cultivated using the modified R2A medium (per L: 175 g NaCl, 20 g MgSO_4_ x 7 H_2_O, 7.88 g TRIS-HCl, 0.17 g yeast extract, 0.17 g tryptone, 0.17 g casamino acids, 0.5 g glucose, 0.5 g soluble stark, 0.3 g K_2_HPO_2_, 0.3 g Na-pyruvate, 3 g Na_3_-citrat x 2 H_2_O, 2 g KCl, 0.2 g CaCl_2_ x 2 H_2_O, 50 ng CuSO_4_ x 5 H_2_O, 4.55 ng (NH_4_)_2_Fe(SO_4_)_2_ x 6 H_2_O, 300 ng MnSO_4_ x H_2_O, 440 ng ZnSO_4_ x 7 H_2_O; CaCl_2_ and trace elements were added after autoclaving). The cultivation took place at 30 °C in the dark on a shaking plate at 120 rpm. Cells were harvested in the mid exponential growth phase (OD_600_ of about 0.4 after 36 h of growth) by centrifugation at 10,000 x g and 18 °C for 10 min. For further experiments, cells were washed with 3 M NaCl, pC_H+_ 6 (corrected pH due to the high ionic strength [21]) three times.

### Uranium(VI) bioassociation

The bioassociation experiments were carried out as described by Bader *et al*. at uranium(VI) concentrations of 10 and 30 μM and a pC_H+_ value of 6 [18]. The uranium used for the experiments was purchased as UO_2_(NO_3_)_2_ x 6H_2_O (remaining stock VKTA, Dresden, Germany) and converted in a muffle furnace at 320 °C to UO_3_ [22]. The resulting uranium(VI) oxide was dissolved in 0.5 M HCl to obtain a stock solution with a concentration of 101 μM of uranium(VI). The suspensions were incubated for different periods of time (0 to 48 h).

Besides the investigation of the kinetics at low and high uranium concentrations, experiments were also conducted on the concentration-dependent association of uranium(VI) (10–60 μM) after 24 hours. All samples were prepared with a biomass concentration of 0.5 mg/mL at a pC_H+_ value of 6 and a sodium chloride concentration of 3 M.

The remaining uranium(VI) content in the supernatants after centrifugation was measured with inductively coupled plasma mass spectrometry (ICP-MS; ELAN 9000, Perkin-Elmer, Waltham, MA, USA). To exclude abiotic uranium(VI) removal from the solution, e.g. due to precipitation and/or chemical sorption to the wall of the vials, samples without cells were treated in the same way. Apart from this, cell samples without uranium(VI) were prepared as a control. All experiments were carried out in triplicates.

### Verification of cell viability

Cells treated under different experimental conditions were washed (3 M NaCl, pC_H+_ 6) and afterwards centrifuged at 10,000 x g and 18 °C for 10 min. The LIVE/DEAD^®^ BacLight^™^ Bacterial Viability Kit (Thermo Fisher Scientific, Waltham, MA, USA) was used for the staining according to the manufacturer’s instructions. The images were taken with the phase-contrast microscope Olympus BX-61 (Olympus Europa Holding GmbH, Hamburg, Germany) using the imaging software “CellSense Dimension 1.11”. Fluorescence was excited by light with wavelengths of 420 nm and 460 nm using the filters Cy3 and FITC.

### *In situ* attenuated total reflection Fourier-transform infrared spectroscopy

The ATR FT-IR spectroscopic association studies were carried out according to Bader *et al*. [18]. The experimental conditions as well as the device parameters were adopted for a better comparability. A uranium(VI) concentration of 30 μM was used for the experiments.

### Time-resolved laser-induced fluorescence spectroscopy

Time resolved laser-induced fluorescence spectroscopic (TRLFS) measurements of supernatants and cell pellets were used to investigate the formed cell-uranium(VI) complexes. TRLFS investigations were performed after 1 h, 4 h, 6 h, 24 h and 48 h of incubation time, except when otherwise stated. For sample preparation, 1 mL of each supernatant was transferred into a semi-micro UV-VIS cuvette. In addition, a sample of the abiotic uranium(VI) solution was prepared the same way for the measurement of a blank spectrum. After sampling, all cuvettes were frozen in liquid nitrogen and stored at -80 °C until measurement. For cell samples, the pellets of the triplicates were washed in 3 M NaCl solution (pC_H+_ 6) and combined to get enough biomass for the special aluminum solid-matter-sample-holders. Subsequent measurements and data evaluation were carried out according to Bader *et al*. [19]. The measurements were performed at a temperature of 150 K for the supernatants and 110 K for the solid samples in order to minimize the quenching effect of the chloride anions on the uranium(VI) fluorescence [23].

In order to enable the assignment of the obtained spectra from the supernatants or cell pellets to different species, new reference substances were examined spectroscopically in addition to compounds already known in literature [19, 24–27]. For this experiment the lipopolysaccharide (LPS) of *Pseudomonas aeruginosa* was used, which is a component of the cell wall of bacteria [28]. Although LPS was not found in archaea, very similar compounds (*e*. *g*. phosphatidylglycerol) can be detected here [16, 28]. The uranium(VI) concentration in the samples was 10 μM, the concentration of LPS 0.5 g/L and a background electrolyte of 3 M NaCl was used. The sample was adjusted to a pC_H+_ value of 6.0 and measured under the same conditions as the samples of the supernatant [19].

Furthermore, for determination of the luminescence spectra of uranyl(VI)-carbonate complexes, reference spectra of uranium(VI) with NaHCO_3_ as a function of the pH value were measured at low temperature, as well. NaHCO_3_ was used to adjust the pH. The samples contained a uranium(VI) concentration of 10 μM at a pH value of 4 to 10 and a background electrolyte of 0.1 M NaClO_4_. The collected spectra were analyzed via parallel factor analysis (PARAFAC) [25]. and the extracted single spectra were compared to the spectra obtained from the bioassociation experiments.

## Results & discussion

### Uranium(VI) bioassociation

**Fig 1:**
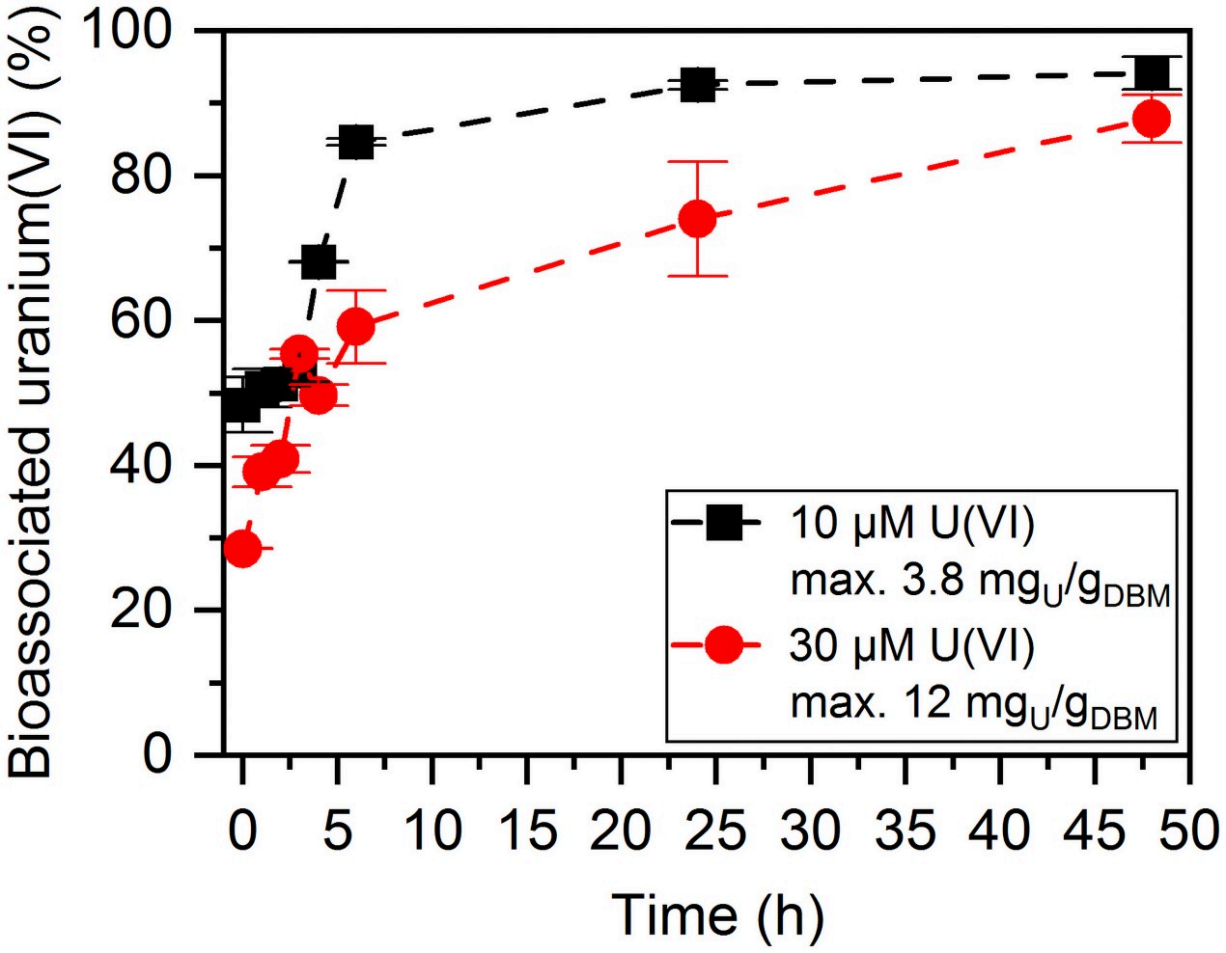
Association of uranium(VI) onto cells of *H*. sp. GP5 1–1 over time. Concentrations of 10 μM (black graph) and 30 μM uranium(VI) (red graph) were used (pC_H+_ 6, DBM = 0.5 mg/mL, [NaCl] = 3 M).

A time-dependent bioassociation experiment was performed to investigate the kinetics of the interactions between *H*. sp. GP5 1–1 and uranium(VI). Two different uranium(VI) concentrations (10 μM and 30 μM) were used. The experiments were performed with a biomass concentration of 0.5 mg/mL at a pC_H+_ of 6 and a sodium chloride concentration of 3 M. Fig 1 shows the curves of the bioassociation of uranium(VI) as a function of time.

At both concentrations the proportion of associated uranium(VI) increases with increasing incubation time. For 10 μM uranium(VI), an association rate of over 80% was achieved after only six hours of exposure. This value increased only slightly until 48 h, reaching a final association rate of 94%. For the conditions investigated (0.5 mg/mL dry biomass (DBM), 48 h) this corresponds to an amount of associated uranium(VI) of approx. 3.8 mg_U_/g_DBM_. This means that after a short exposure time, an equilibrium of the association process to the cells of the archaeon is reached. Already at the beginning of the process (resuspension of the pellet in uranium(VI) solution and subsequent centrifugation) a relatively high proportion of bioassociated uranium(VI) of almost 50% occurred. This process can be classified as biosorption. Therefore, during the first three hours of incubation the proportion remained almost constant. Afterwards, different metabolic processes can take place leading to a further increase in the amount of associated uranium(VI).

In contrast, the bioassociation process is significantly slower at a uranium(VI) concentration of 30 μM. An equilibrium is reached after 24 h. After only one hour of exposure, about 40% of the uranium(VI) present in the solution was associated onto the cells. After 48 h, an association rate of almost 90% was achieved, although the curve flattens off considerably during longer exposure times due to the equilibrium conditions. In this case, approx. 12 mg_U_/g_DBM_ are achieved. A tripling of the concentration thus leads to a three times higher loading with uranium(VI). This shows that saturation does not occur in this concentration range. In comparison, under similar conditions (40 μM uranium(VI), 0.5 mg/mL DBM, 3 M NaCl solution at pC_H+_ 6) the very closely related microorganism *Halobacterium noricense* DSM 15987^T^ shows equal association values for uranium(VI) of 9.3 ± 0.4 mg_U_/g_DBM_ [18]. In contrast, the sorption capacity of the halophilic bacterium *Brachybacterium* sp. G1 is significantly higher with approx. 971 ± 29 mg_U_/g_DBM_ after 24 h of incubation (40 μM uranium(VI), 0.075 mg/mL DBM, 1.7 M NaCl solution at pC_H+_ 6) [17]. An explanation for these high association values for *Brachybacterium* sp. G1 is the high number of carboxylate groups within the peptidoglycan layer of the cell wall [29].

A simple biosorption process is characterized by a quick bound of the sorptive (uranium(VI)) to the sorbent (microorganism) within a few hours [5, 30]. This means that a pure biosorption did not occur at any concentration. At both concentrations the biosorption process to functional groups on the cell surface is the first step, which is in general a very fast process (approx. 0–2 h). Biosorption is independent of the cell metabolism, hence it is a passive process and both living and dead cells should be able to [5, 30]. Afterwards, the amount of cell-bound uranium(VI) is still increasing, but to a lesser extent. One reason for this may be further association processes, which are supported by partial cell lysis as a result of dying cells or the formation of biominerals.

The very closely related halophilic archaeon *Halobacterium noricense* DSM 15987^T^ exhibits a much more complex, multi-step association process with uranium(VI). Its interactions with this radionuclide have already been investigated by Bader *et al*. [17–19]. Both microorganisms differ only in one base pairing of 818 bp of the 16S rRNA gene sequence. The used experimental conditions were very similar (40 μM uranium(VI), 0.5 mg/mL DBM, 3 M NaCl solution at pC_H+_ 6). Despite the close relationship between the two archaea, clear differences in the bioassociation behavior of uranium(VI) to the cells are obvious. In the case of *H*. *noricense* DSM15987^T^ a multiphase association process occurred. The microorganism showed a rapid association of uranium(VI) to the cells first, followed by a desorption of the actinide. Afterwards, a slow association occurred, which was only completed after about two weeks. However, at higher uranium(VI) concentrations (85 μM), no desorption phase took place either [18, 19]. It can be concluded that microorganisms which belong to the same genus or even species, do not always show the same interaction mechanisms under similar experimental conditions. Therefore, a more detailed study of the individual genera and species is indispensable.

### Cell viability during uranium(VI) exposure

**Fig 2:**
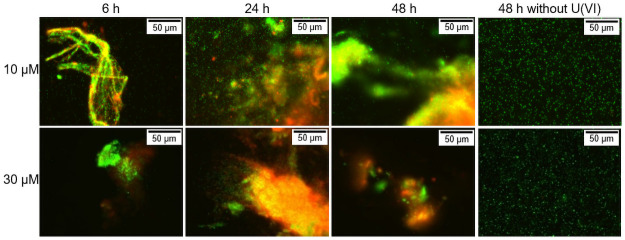
Live/Dead staining of the cells of *H*. sp. GP5 1–1 after different incubation times. Cells were dyed with LIVE/DEAD^®^ BacLight^™^ Bacterial Viability Kit; green = living cells, red = dead cells.

The live/dead staining images show the formation of cell agglomerates after six hours of incubation at both concentrations (Fig 2). During this agglomeration, organic molecules having a huge number of functional groups are probably released by the cells. One of these components, which has already been reported in the literature, could be *N*-acetylneuraminic acid [31]. Together with extracellular genomic DNA (eDNA), these glycoproteins are a major component of extracellular polymeric substances, which are essential in microbial biofilms as described by Fröls *et al*. [31]. This may cause a further increase in the amount of bioassociated uranium(VI) due to additional available functional groups of these compounds. As a result, the dissolved uranium(VI) was almost completely associated onto the biomass. After longer incubation times some of the cells died. As a result of the subsequent lysis of the cells, an increase in functional groups available for the bioassociation was also conceivable. The formed agglomerates had almost the same size at both uranium(VI) concentrations. However, the proportion of living cells was significantly higher at 10 μM than at 30 μM uranium(VI). Furthermore, at the lower uranium(VI) concentration, suspended cells were still present next to the agglomerates up to approx. 6 h. Nevertheless, this relatively small amount of uranium(VI) had already a clear influence on the cells. At both concentrations the proportion of dead cells increased with increasing incubation times. Fig 2 also shows that cells incubated without uranium(VI) did not agglomerate even after 48 h and showed a high viability even after this time.

### Spectroscopic investigations of the bioassociation process

*In situ* ATR FT-IR spectroscopy allows us to detect to which functional groups uranium(VI) binds. Typical binding or complexing partners for uranium(VI) on the surface of microorganisms are carboxylate, phosphoryl, amino or even hydroxyl groups [32, 33].

For the detection of bioassociated species by *in situ* ATR FT-IR spectroscopy, it is necessary to prepare an archaeal film as stationary phase directly on the ATR crystal. In the following, this film is rinsed with aqueous background and uranium(VI) solutions at identical pH and ionic strength. Changes on this microbial film are monitored by recording single beam spectra continuously every 30 seconds. By calculation of difference spectra, only changes upon uranium(VI) interaction with the microorganisms are observed, constant spectral parts (characteristic bands from the archaeal film, the background, the spectrometer) are not displayed in the difference spectra.

**Fig 3:**
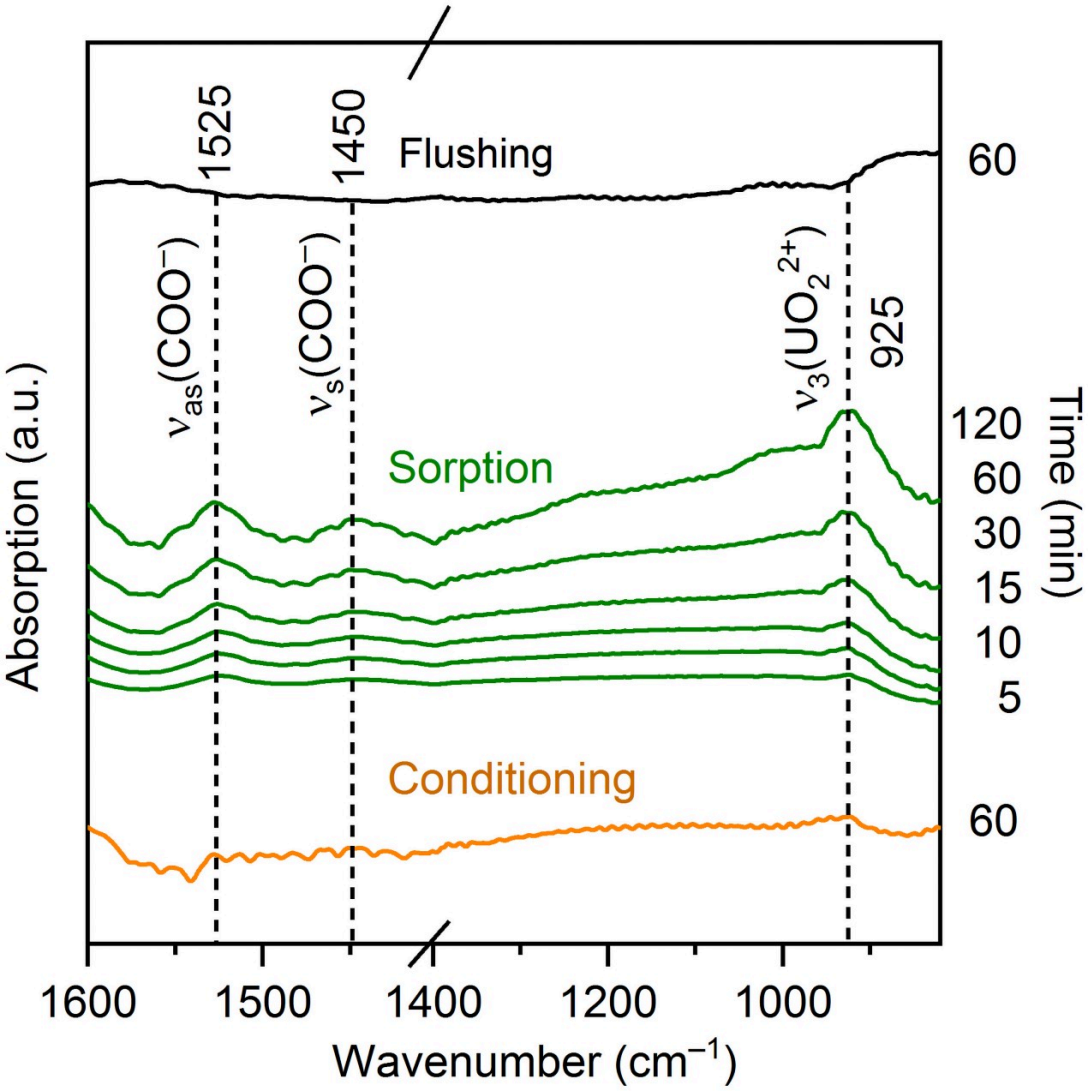
*In situ* ATR-FT-IR difference spectra of uranium(VI) association on *H*. sp. GP5 1–1 cells. (30 μM U(VI), pC_H+_ 6.0, [NaCl] = 3 M).

In a first step, this cell film needs to be equilibrated to the sample conditions. This can be performed by rinsing the background electrolyte (3 M NaCl solution, pC_H+_ 6.0) over the film for a prolonged time period (1 h), which is called conditioning. Fig 3 (orange trace) shows during conditioning no bands in the graph except for the water band at a wavenumber of approx. 1630 cm^−1^. This is a measure of quality for the stability of the archaeal film.

Exposure to uranium(VI) resulted in significant changes in the spectral regions around 1400–1550 cm^−1^ and 850–950 cm^−1^ (green traces). The intensities of the bands increased with increasing exposure time (up to 120 minutes). This suggests an association of uranium(VI) onto the cells of the archaeal film. The band at 925 cm^−1^ can be attributed to the asymmetric stretching vibrational mode of the uranyl(VI) moiety, ν_3_(UO_2_^2+^).

Generally, the frequency of this mode that is observed at 961 cm^−1^ at a fully hydrated state in highly acidic aqueous solution [34], is shifted to lower frequencies upon complexation in solution [35] and at biogeochemical surfaces [18, 36]. This shift of the uranyl(VI) band to 925 cm^−1^ is characteristic for complexation to carboxylate groups. This is in accordance with the spectral signature, we observe at higher wavenumbers. The bands at 1450 cm^−1^ and 1525 cm^−1^ can be assigned to the symmetrical ν_s_(COO^−^) and asymmetrical ν_as_(COO^−^) stretching vibrations of the carboxylate group [37, 38]. After 120 minutes no further increase of the band intensities was observed, indicating that an equilibrium was reached at the archaeal film. Moreover, the absence of further bands or shifting of the ν_3_(UO_2_^2+^) mode hints that no interaction with other cell functional groups, such as phosphoryl groups occurred under the given conditions [37].

As a final step, the archaeal film was rinsed again with the background electrolyte. The calculated difference spectrum (black trace) shows no significant bands at the corresponding wavenumbers. As a conclusion, during one hour only a very small amount of uranium(VI) was removed from the cells.

A comparison with the very closely related *H*. *noricense* DSM15987^T^ shows significant differences in uranium(VI) complexation. For this microorganism, the association occurred via carboxylate as well as phosphoryl groups, as demonstrated by *in situ* ATR FT-IR-spectroscopy [17, 18]. In contrast to the similar amounts of biassociated uranium(VI), this also shows that different interaction mechanisms occur by the two microorganisms. For *Brachybacterium* sp. G1, however, ATR FT-IR spectroscopy shows the binding of uranium(VI) onto the cells only via carboxylate groups, as well [17].

### Luminescence spectroscopic studies of the bioassociation

**Fig 4:**
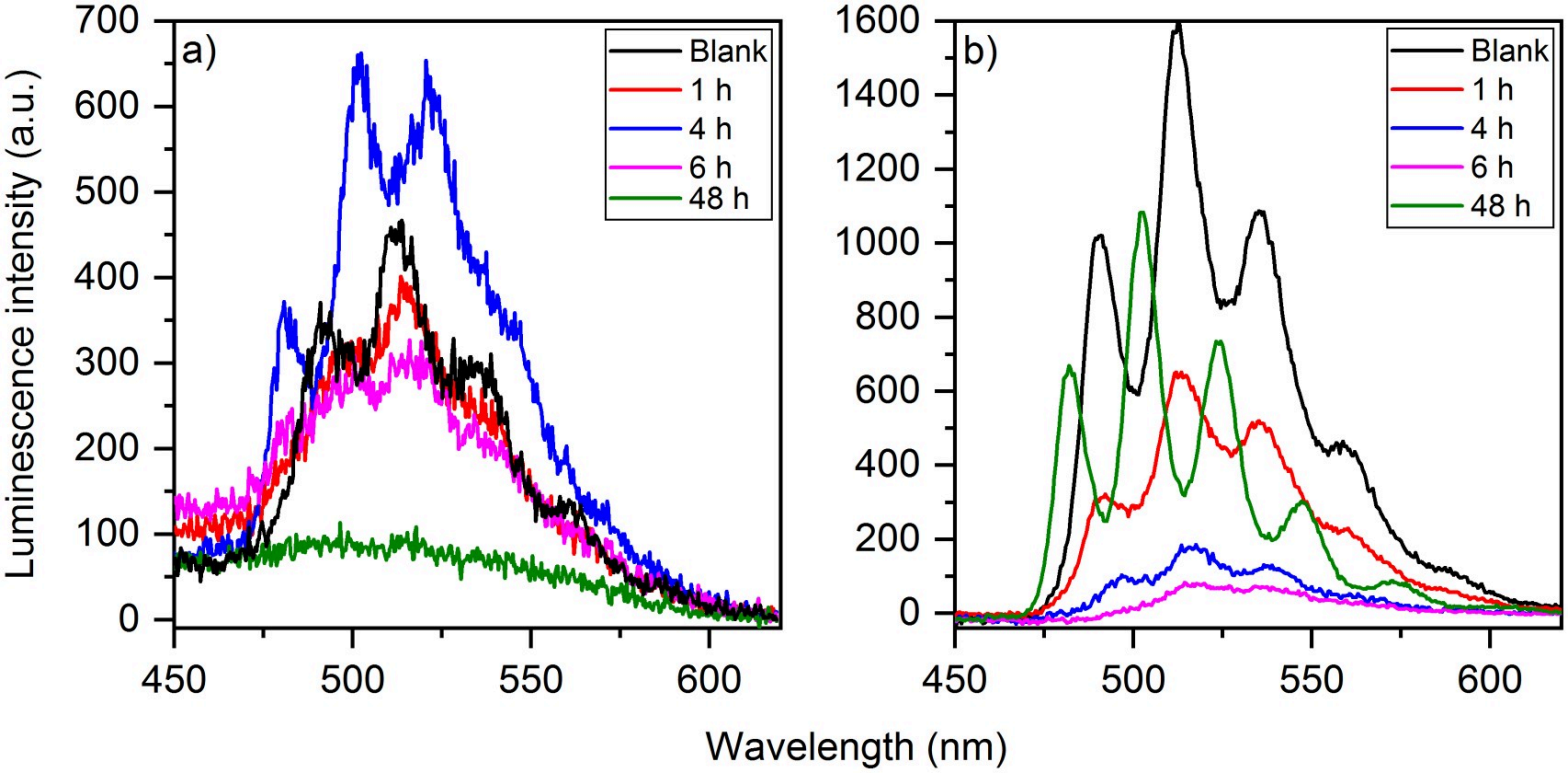
Emission spectra of the supernatants after different incubation times, (1 h– 48 h) for an initial uranium(VI) concentration of (a) 10 μM and (b) 30 μM.

At selected stages of the sorption experiment, time-resolved laser-induced fluorescence spectroscopic studies were performed on both washed cells and supernatants (Fig 4).

Fig 4 shows the emission spectra of the supernatants after different incubation times for the initial uranium(VI) concentrations of 10 μM and 30 μM. At the concentration of 10 μM the luminescence intensity increased until 4 h and a new species occurred what can also be seen from a shift in the peak positions. After 48 h almost the entire amount of uranium(VI) was associated onto the cells. Therefore, no spectrum could be recorded.

At the higher uranium(VI) concentration the luminescence intensity decreased with increasing incubation time of 1 h to 6 h. In addition, a new species was formed, which was observed especially in the spectrum after 48 h with an increasing intensity and a shift of the bands to smaller wavelengths. Changes to the luminescence spectrum of the abiotic blank of the original uranium(VI) solution occurred at both concentrations, as well (Fig 4).

**Fig 5:**
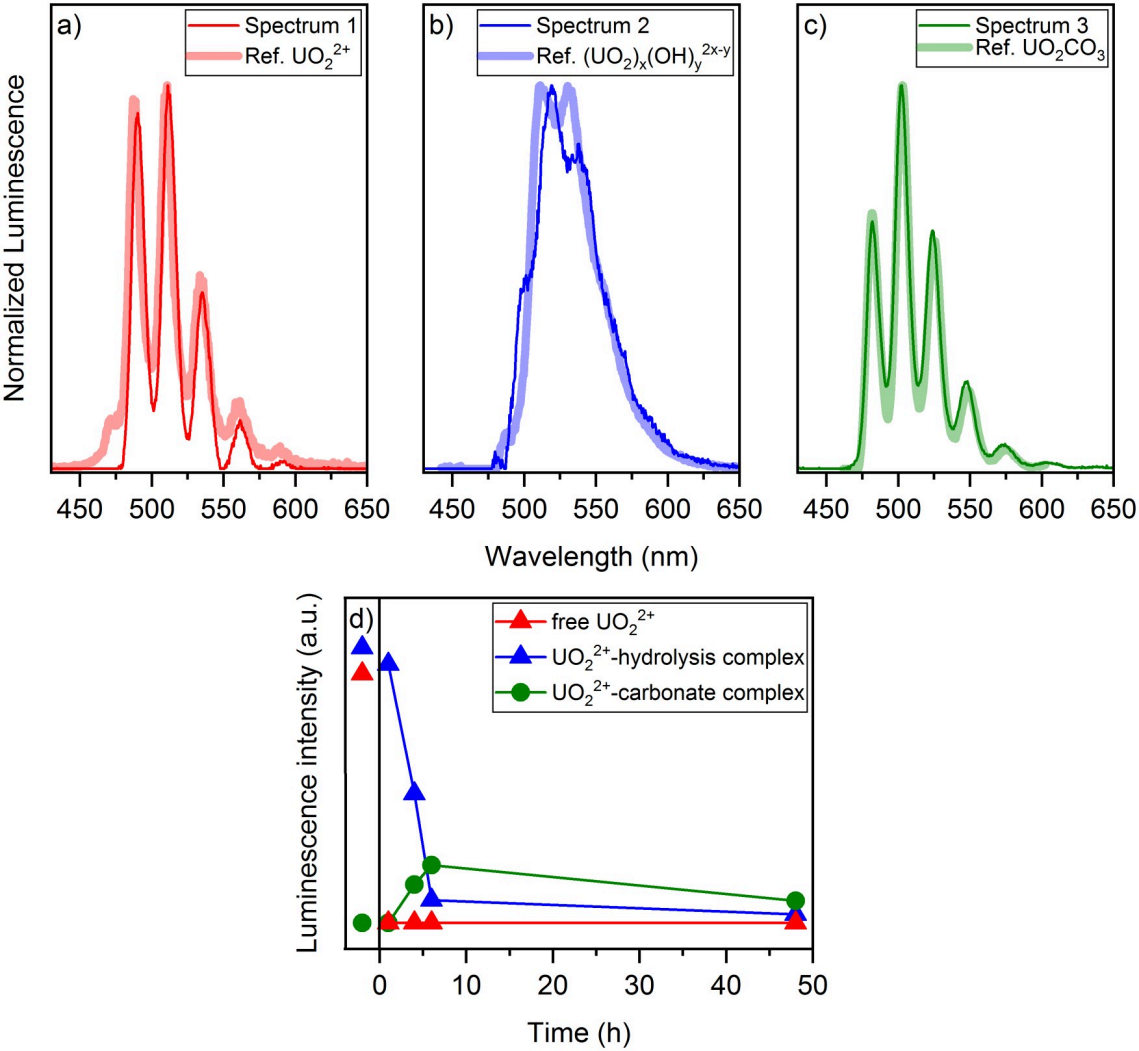
Extracted spectra and distribution of the aquatic uranium(VI) species at 30 μM uranium(VI). Spectra extracted using PARAFAC of the time-resolved emission spectra of the supernatants compared with the reference spectra of (a) free uranyl(VI) cation, (b) uranyl(VI)-hydrolysis complex, (c) uranyl(VI)-carbonate complex; (d) species distribution of the aquatic species as a function of the incubation time under consideration of the bioassociation (red = free uranyl(VI), blue = uranyl(VI)-hydrolysis complex, green = uranyl(VI)-carbonate complex).

Due to the partial spectral superposition, it is not possible to draw direct conclusions from the measured emission spectra about the uranium(VI) species present in the supernatants. In order to obtain the individual spectra of the various species involved, a peak deconvolution was carried out using PARAFAC [25]. The spectra of three different species could be extracted at a uranium(VI) concentration of 30 μM (Fig 5A–5C).

By using reference spectra a partial assignment of the spectra to different uranyl(VI) compounds was possible. The band positions of extracted spectra and references are shown in Table 1. The extracted spectrum 1 can be assigned to the free uranyl(VI). Spectrum 3 clearly shows the structure of the 1:1-uranyl(VI)-carbonate complex being the dominating uranyl(VI)-carbonate species under these conditions [39]. Spectrum 2 is the only one which could not be clearly assigned to a specific complex. A comparison with the spectrum of the 3:5-uranyl(VI)-hydrolysis complex, (UO_2_)_3_(OH)_5_^+^ shows a partial agreement, but this species can be another polynuclear hydroxo-complex, as well [25, 26].

**Table 1:** Assignment of the band positions of the extracted time-resolved laser-induced fluorescence spectra. Extraction of the spectra was performed using PARAFAC.

	Band positions (nm)	Reference
a) Supernatants at 30 μM uranium(VI).		
Spectrum 1 supernatant	490.3 511.1 535.2 561.5 591.5	This work
UO_2_^2+^	488.5 510.7 534.8 560.7 588.9	This work
Spectrum 2 supernatant	499.1 519.0 538.4 561.0 590.1	This work
(UO_2_)_3_(OH)_5_^+^	496.0 511.0 533.0 557.0 584.0	[25, 26]
Spectrum 3 supernatant	481.9 502.3 524.0 547.2 574.5	This work
UO_2_CO_3_	482.0 502.9 525.6 549.2 575.1	This work
b) Cell pellets at 30 μM uranium(VI).		
Spectrum 1 pellet	492.3 525.2 546.8 573.2 600.8	This work
Poly-Carbonate	528.3 554.2	[27]
Spectrum 2 pellet	498.4 523.1 540.9 570.1	This work
Lipopolysaccharide	495.0 517.8 539.9 566.4 592.8	This work
(UO_2_)_3_(PO_4_)_2_ ⋅ 4H_2_O	495.0 518.2 541.3 571.3 594.0	[27]
c) Supernatants at 10 μM uranium(VI).		
Spectrum 3 supernatant	497.7 518.1 537.5 563.9 591.9	This work
Lipopolysaccharide	495.0 517.8 539.9 566.4 592.8	This work
(UO_2_)_3_(PO_4_)_2_ ⋅ 4H_2_O	495.0 518.2 541.3 571.3 594.0	[27]

The species distribution of the three different species is shown in Fig 5D. In the blank solution of 30 μM uranium(VI) in 3 M NaCl only the free uranyl(VI) cation and the uranyl(VI)-hydrolysis complex are present in almost equal parts. Already after one hour the whole proportion of the free uranyl(VI) is associated to the biomass. Therefore, the free uranyl(VI) is the first species to bind to the cells. In contrast, the hydrolysis complex (species 2) decreases slower with increasing incubation time. After one hour the largest proportion remains still in the supernatant. After four hours a new species occurs which can be assigned to the uranyl(VI)-carbonate complex.

It is possible that carbon dioxide is microbially released by the cells of the archaeon. The resulting carbonate strongly complexes the uranyl(VI) ion and thus forms the uranyl(VI)-carbonate complex [40]. Therefore, the third species could only be detected after a few hours of incubation. After 48 h the proportion of this species decreases again due to the ongoing bioassociation. The proportion of the hydrolysis complex is almost zero by end of the experiment.

**Fig 6:**
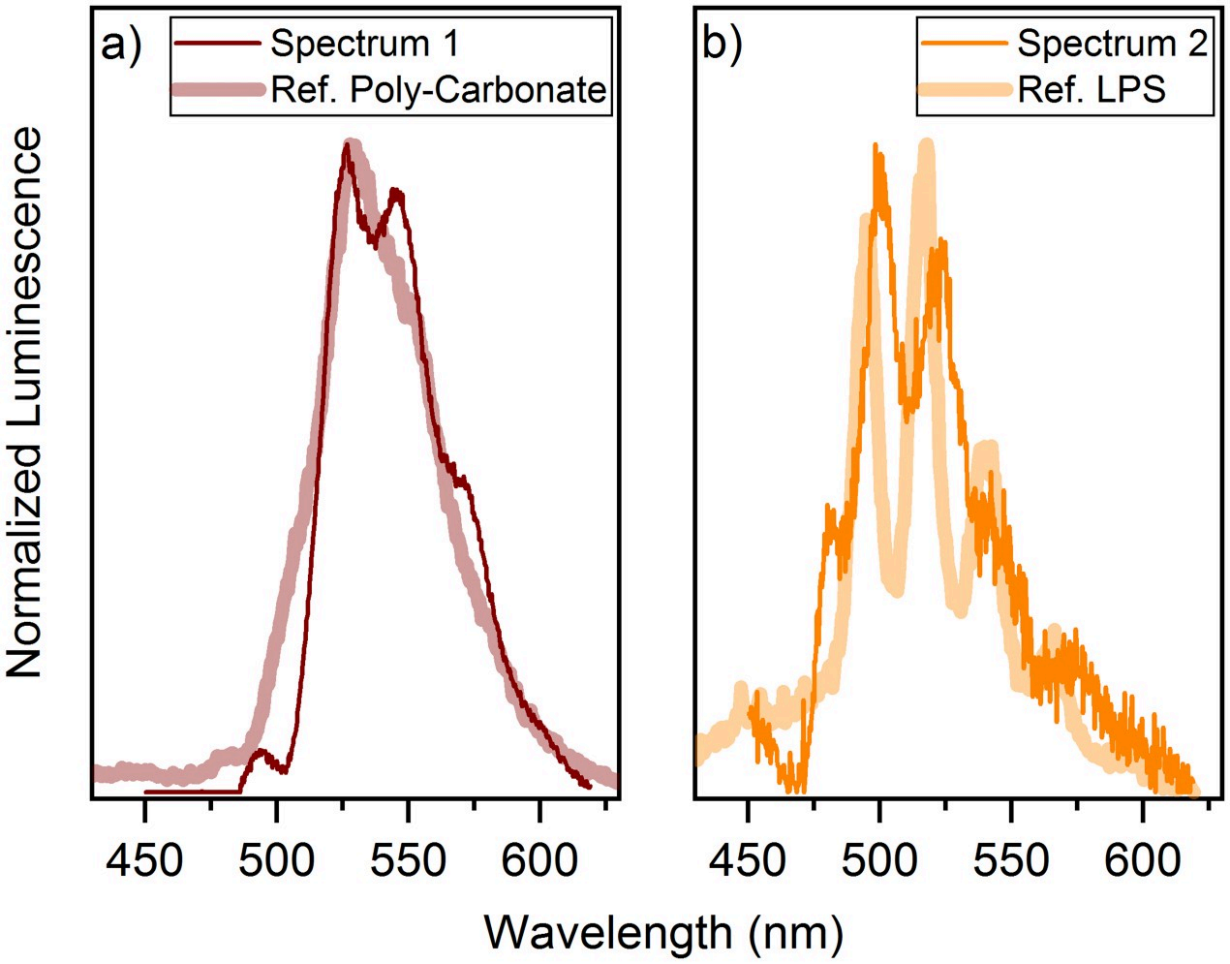
Extracted spectra of the uranium(VI) species in the cell pellets of the uranium(VI) association experiment. Spectra extracted using PARAFAC of the time-resolved emission spectra of the cell pellets at 30 μM uranium(VI) compared with the reference spectra of (a) poly-carbonate and (b) lipopolysaccharide (LPS).

The luminescence spectra of the cell pellets have also been evaluated using PARAFAC [25]. According to the extraction, two uranium(VI) species occur in the solid samples (Fig 6):

In order to assign the extracted spectra more precisely to different species, they were compared with reference spectra. Spectrum 1 shows a clear similarity with a carboxylate species. Spectrum 2 has a phosphate structure, as can be seen by the comparison with the spectrum of lipopolysaccharide (LPS) (see Fig 6) and by the assignment of the band positions of the extracted species with reference compounds as e.g. (UO_2_)_3_(PO_4_)_2_ ⋅ 4H_2_O (see Table 1B). The band positions and the spectral decomposition do not fit exactly but a rough classification to these species is nevertheless possible. LPS has mainly phosphate groups. It is not a component of the archaeal cell wall, but there are structurally similar phosphate compounds, such as phosphatidylglycerol [16, 28].

In the species distribution of the cell pellets (S2 Fig) only the carboxylate species is present at the beginning of the association process. This species predominates during the entire process. The phosphate species, on the other hand, can only be observed to a lesser extent.

It is possible that the association of uranium(VI) occurs first to the carboxylate groups and afterwards, a binding to the phosphate groups or the formation of a phosphate complex outside the cells occurs (possible biomineralization). During longer incubation periods, the release of organic compounds from the cells takes place, as well as an increase in the functional groups due to the partial death and subsequent lysis of the cells [31]. This results in a further increase in the amount of cell associated uranium(VI). It is possible that the number of carboxylate groups also outweighs the number of phosphate groups in these discharged compounds, which explains the high proportion of the carboxylate species in comparison to the phosphate species (see section 3.5 Concentration-dependent experiment).

However, no binding of uranium(VI) to phosphate groups in the ATR FT-IR spectrum of *H*. sp. GP5 1-1 was observed. But, *in situ* ATR FT-IR spectroscopy covers only the first two hours of the bioassociation process. It’s possible that a binding to phosphate groups did not occur during this *in situ* set-up.

Another possible explanation for not observing the binding to phosphate groups in the ATR FT-IR spectrum would be the formation of phosphate minerals outside the cells as a kind of biomineralization. The band positions of the extracted TRLFS species also show a certain agreement with these compounds (*e*. *g*. (UO_2_)_3_(PO_4_)_2_ ⋅ 4H_2_O).

During the bioassociation experiment with 10 μM uranium(VI), only luminescence spectra of the supernatants were recorded. In addition to the free uranyl(VI) and the carbonate complex, PARAFAC [25] analysis revealed a further spectrum. A comparison of the band positions with different references shows a possible assignment to an aquatic phosphate species (see Table 1C and S3A Fig). The species distribution also shows a relatively high proportion of the complex during the whole incubation time (S3B Fig). This could be another incidence for an occurring biomineralization by cell-released phosphate species at lower uranium(VI) concentrations. The hydrolysis species on the other hand could not be observed. This again indicates the clear differences between the association processes at 10 μM and 30 μM uranium(VI).

### Concentration-dependent experiment

Besides the investigations regarding the association kinetics, a concentration-dependent experiment with uranium(VI) concentrations between 10 μM and 60 μM was performed. All these experiments were carried out for 24 h with a biomass concentration of 0.5 mg/mL at a pC_H+_ value of 6 and a sodium chloride concentration of 3 M.

The results of this experiment show a linear correlation between the uranium(VI) concentration and the amount of bioassociated uranium(VI) per dry biomass (S4 Fig). No saturation occurred, not even at a concentration of 60 μM. At this concentration approx. 23 mg_U_/g_DBM_ are achieved. With increasing concentrations, the size of the agglomerates and the proportion of dead cells increased (S5 Fig). Experiments with higher uranium(VI) concentrations were not carried out because above 60 μM uranium(VI) cells form large agglomerates within a short time containing a high percentage of dead cells.

To investigate the concentration-dependent bioassociation process more in detail, luminescence spectroscopic investigations were performed, as well. Using parallel factor analysis, two spectra could be extracted from the luminescence spectra of the supernatants as well as two from those of the cell pellets. The aquatic species can be assigned to the uranyl(VI)-carbonate and the uranyl(VI)-hydrolysis complex. The free uranyl(VI) could not be detected in the supernatants due to the long incubation time. In the cell pellets, a uranium(VI)-carboxylate and a uranium(VI)-phosphate species can be observed again due to the comparison with the references (Table 2; spectra see S6 and S7 Figs).

**Table 2:** Assignment of the band positions of the extracted time-resolved laser-induced fluorescence spectra from the concentration-dependent bioassociation experiment. Extraction of the spectra was performed using PARAFAC.

	Band positions (nm)	Reference
a) Supernatants		
Spectrum 1 supernatant	497.8 510.7 533.8 556.6	This work
(UO_2_)_3_(OH)_5_^+^	496.0 511.0 533.0 557.0 584.0	[25, 26]
Spectrum 2 supernatant	481.4 501.7 523.9 546.9 571.9	This work
UO_2_CO_3_	482.0 502.9 525.6 549.2 575.1	This work
b) Cell pellets		
Spectrum 1 pellet	528.2 554.5 571.0 597.1	This work
Poly-Carbonate	528.3 554.2	[27]
Spectrum 2 pellet	495.3 518.1 536.2 568.5 594.6	This work
Lipopolysaccharide	495.0 517.8 539.9 566.4 592.8	This work
(UO_2_)_3_(PO_4_)_2_ ⋅ 4H_2_O	495.0 518.2 541.3 571.3 594.0	[27]

**Fig 7:**
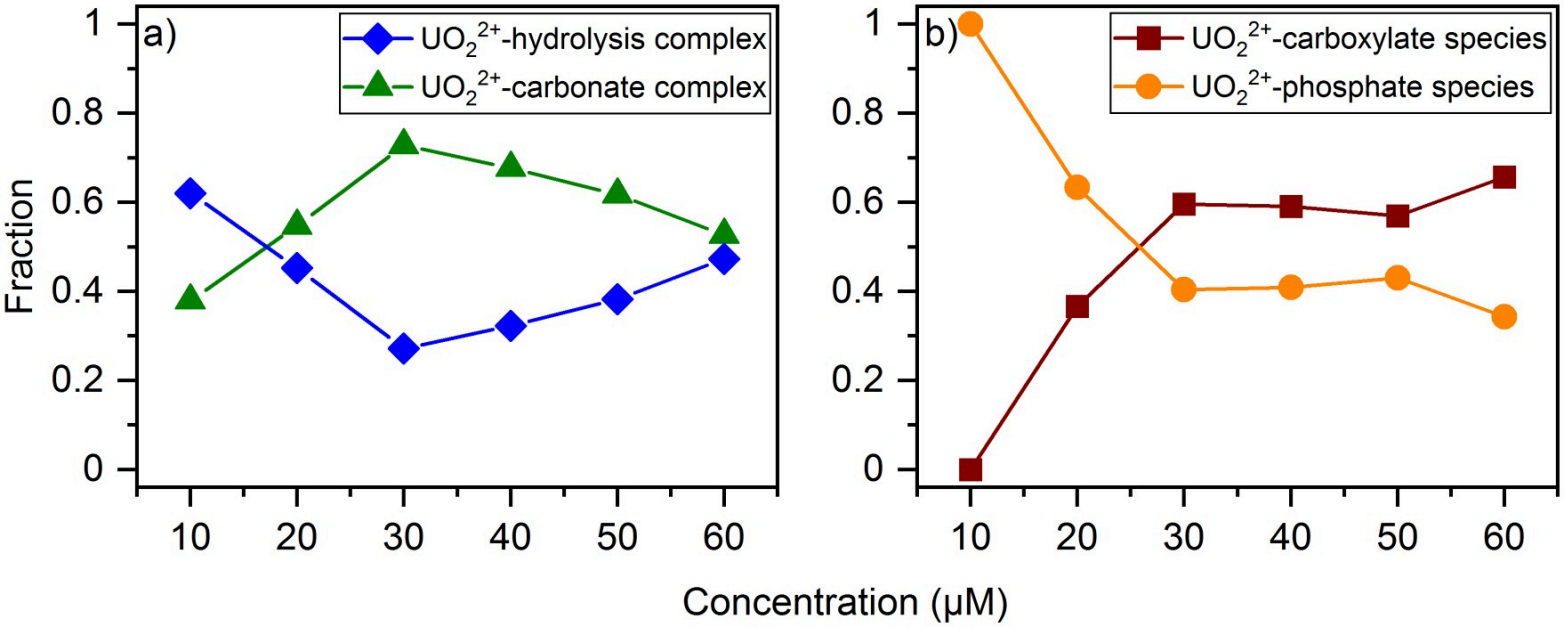
Species distribution in supernatants and cell pellets of the concentration-dependent experiment. Based on the normalized luminescence intensities as a function of the uranium(VI) concentration in the (a) supernatants (blue = uranyl(VI)-hydrolysis complex, green = uranyl(VI)-carbonate complex) and (b) cell pellets (brown = uranium(VI)-carboxylate species, orange = uranium(VI)-phosphate species).

The species distribution of the aquatic species (Fig 7A) shows at low concentrations an increase in the carbonate species until 30 μM. Afterwards, this proportion decreases. The distribution of the uranyl(VI) hydrolysis complex is exactly the opposite. The amount of uranium(VI) available for the bioassociation increases with concentration. Therefore, up to 30 μM uranium(VI) a larger amount of the carbonate complex can be formed. At higher uranium(VI) concentrations, however, more and more cells of *H*. sp. GP5 1 1 die because of its toxicity. This reduces the microbial activity and less carbon dioxide could be released by the microorganisms, which results in a reduced proportion of the carbonate species.

The species distribution in the cell pellets shows a decreasing proportion of the phosphate species with increasing uranium(VI) concentration (Fig 7B). In contrast, the proportion of the carboxylate species increases. The phosphate species is the only species that occurs at a uranium(VI) concentration of 10 μM. However, their proportion decreases to 30% at a uranium(VI) concentration of 60 μM. This suggests that potential biomineralization occurs primarily at lower uranium(VI) concentrations.

The proportions of the two species at 30 μM differ slightly from the distribution in the time-dependent experiment with 30 μM uranium(VI) solution after 24 h, with the carboxylate species dominating in both cases. Due to an increased excretion of organic compounds from the cells with increasing uranium(VI) concentration, as well as cell lysis as a result of cell death, more carboxylate groups are available for the association of uranium(VI).

A comparison to the closely related *Halobacterium noricense* DSM 15987^T^ shows a certain agreement in the interaction mechanisms at different uranium(VI) concentrations. At 30 μM uranium(VI) a uranyl(VI) phosphate species is predominant in the spectra of the cell pellets, whereas at 85 μM the binding of uranium(VI) onto the cells occurs primarily via carboxylate groups of a lactate binding motive [19]. Nevertheless, these processes take place at higher uranium(VI) concentrations than for the studied *Halobacterium* species. This is a significant difference between the two microorganisms.

### Comparison between *H*. sp. GP5 1–1 and *H*. *noricense* DSM 15987^T^

At first glance, the interaction mechanisms of the two halophilic archaea with uranium(VI) appear to be very different. However, if a look is taken at different concentrations, some commonalities can certainly be found. These similarities and differences are summarized in Table 3.

**Table 3:** Comparison of the interactions with uranium(VI) between *H*. sp. GP5 1–1 and *H*. *noricense* DSM 15987^T^.

	*H*. sp. Gp5 1–1	*H*. *noricense* DSM 15987^T^
Association kinetics:	10 μM and 30 μM U(VI): multistage process without desorption	a) 30 μM U(VI): multistage process with desorption [17–19]
		b) 85 μM U(VI): multistage process without desorption [19]
Involved functional groups on the cell surface in the binding of uranium(VI)	Carboxylate groups	Carboxylate and phosphoryl groups [17, 18]
Cell reaction to uranium(VI) incubation	a) 10 μM U(VI): biomineralization	a) 30 μM U(VI): biomineralization [19]
	b) 30 μM U(VI): biofilm formation	b) 85 μM U(VI): biofilm formation [19]

At a uranium(VI) concentration of 30 μM *H*. *noricense* DSM 15987^T^ shows a multistage association process including a desorption step [17–19]. Furthermore, after longer incubation times a biomineralization occurs including a uranium(VI) phosphate species [19]. At the same concentration, *H*. sp. GP5 1–1 exhibit also a multistage association process but no desorption occurs. Uranium(VI) is bound predominantly to biofilm-like structures containing mostly carboxylate groups. These biofilm-like structures can be observed in *H*. *noricense* DSM 15987^T^, as well. However, the uranium(VI) concentrations at which these structures occur are significantly higher with 85 μM [19]. Such similarities can also be found at even lower concentrations for *H*. sp. GP5 1–1. At 10 μM uranium(VI) this halophilic archaea shows a biomineralization of uranium(VI) also under participation of phosphates. In summary, the interaction mechanisms of both halophilic microorganisms are more similar than initially suspected. However, the concentration ranges in which the different processes take place are very different between the two archaea. This also explains a different tolerance of both microorganisms to uranium(VI).

## Conclusions

Investigations of the interactions of naturally occurring microorganisms in the host rock formations with radionuclides are necessary for an overall concept of the safety of a nuclear repository. In this study, kinetics of the bioassociation of uranium(VI) onto cells of the isolated halophilic archaeon *Halobacterium* sp. GP5 1–1 showed a different behavior in dependence on the uranium(VI) concentration. However, at both investigated concentrations (10 and 30 μM) the occurring process is more complex and not only a simple biosorption.

Different spectroscopic techniques, as well as microscopy provided a deeper process understanding of the interactions between uranium(VI) and *Halobacterium* sp. GP5 1–1. Cell agglomerates occurred independently of the uranium(VI) concentration after similar incubation times. Using spectroscopic methods, a cell-related binding of uranium(VI) via carboxylate groups was proven. Additionally, a phosphate species was observed, dominating predominantly at lower uranium(VI) concentrations.

In the supernatants, we detected the formation of a uranyl(VI)-carbonate complex, presumably due to microbial released carbon dioxide. At lower uranium(VI) concentrations, the formation of a phosphate species in solution was detected, as well.

**Fig 8:**
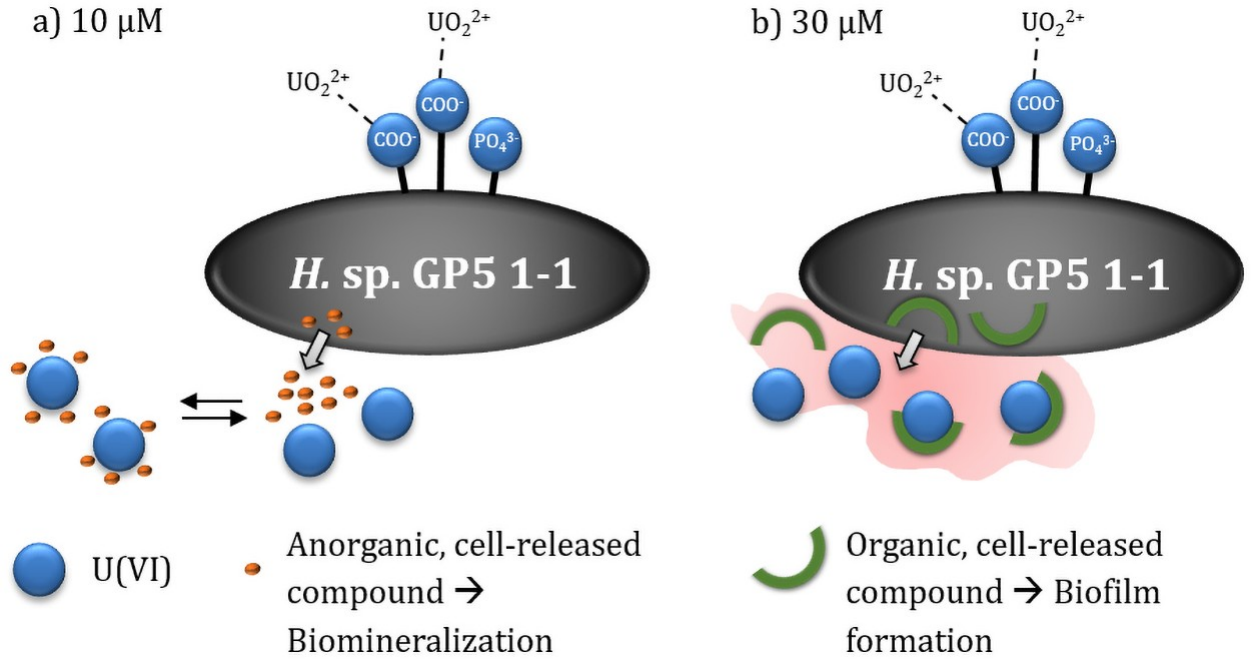
Summary of the dominant interaction processes of *H*. sp. GP5 1–1 with uranium(VI). Based on the two investigated uranium(VI) concentrations of a) 10 μM and b) 30 μM.

The samples show evidence of an occurring biomineralization with participation of cell-released phosphate at lower uranium(VI) concentrations outside the cells. At higher uranium(VI) concentrations, on the other hand, there is a binding of uranium(VI) to carboxylate groups of biofilm-like structures. Both processes will lead to an immobilization and thus to a retention of uranium(VI) in rock salt. Fig 8 shows a summary of the occurring interaction mechanisms at 10 and 30 μM uranium(VI).

Furthermore, the study shows differences in the interaction mechanisms compared to the very closely related *Halobacterium noricense* DSM 15987^T^. Similar interaction mechanisms occur in this microorganism depending on the uranium(VI) concentration, but the concentration ranges differ significantly [19]. Therefore, it is crucial for the long-term safety of a nuclear repository to investigate the influence of different microorganisms present in the host rock, even of the same genus. Only in this way is it possible to develop a comprehensive safety concept, for which this study made an important contribution. Since the interactions of this microorganism with uranium(VI) are now well understood, further studies regarding the influence of other radionuclides present in high-level radioactive waste would also contribute to this safety assessment.

## Supporting information

S1 Dataset 16S rRNA gene sequence of *Halobacterium* sp. GP5 1–1. (DOCX)

S1 Fig Phylogenetic dendrogram (neighbor-joining method) of *Halobacterium* sp. GP5 1–1 and its closest phylogenetic relatives. Based on an alignment of 16S rRNA gene sequences (aligned with ClustalX-MEGA 6.06). GenBank accession numbers are shown in brackets.
(DOCX)

S2 Fig Species distribution in cell pellets of the uranium(VI) association experiment. Based on the normalized luminescence intensities as a function of the incubation time at 30 μM uranium(VI) (brown = uranium(VI)-carboxylate, orange = uranium(VI)-phosphate).
(DOCX)

S3 Fig Extracted spectrum 3 and distribution of the aquatic uranium(VI) species at 10 μM uranium(VI). (a) Spectrum 3 extracted using PARAFAC of the time-resolved emission spectra of the supernatants compared with the reference spectrum of lipopolysaccharide (LPS); (b) species distribution of the aquatic species as a function of the incubation time at 10 μM uranium(VI) under consideration of the bioassociation (red = free uranyl(VI), green = uranyl(VI)-carbonate complex, orange = uranyl(VI)-phosphate complex); in the sample after 48 h no uranium(VI) was still detectable.
(DOCX)

S4 Fig Concentration-dependent association of uranium(VI) onto *H*. sp. GP5 1–1. (t = 24 h, DBM = 0.5 mg/mL, [NaCl] = 3 M).
(DOCX)

S5 Fig Live/Dead staining of the cells of *H*. sp. GP5 1–1 at different uranium(VI) concentrations. Cells were dyed with LIVE/DEAD^®^ BacLight^™^ Bacterial Viability Kit; green = living cells, red = dead cells (after 24 h of incubation).
(DOCX)

S6 Fig Extracted spectra of the uranium(VI) species in the supernatants of the concentration-dependent experiment. Spectra extracted using PARAFAC of the time-resolved emission spectra compared with the reference spectra of (a) uranyl(VI)-hydrolysis complex, (b) uranyl(VI)-carbonate complex.
(DOCX)

S7 Fig Extracted spectra of the uranium(VI) species in the cell pellets of the concentration-dependent experiment. Spectra extracted using PARAFAC of the time-resolved emission spectra compared with the reference spectra of uranium(VI) bound to a) poly-carbonate and b) lipopolysaccharide (LPS).
(DOCX)
