# Supplementary material for: Microscopic and spectroscopic bioassociation study of uranium(VI) with an archaeal Halobacterium isolate
Source: PLoS One. 2022 Jan 13;17(1):e0262275. doi: 10.1371/journal.pone.0262275 (PMC8757991; doi:10.1371/journal.pone.0262275)
Supplement: S1 Dataset — (DOCX) [file pone.0262275.s001.docx]

**S1 Dataset. 16S rRNA gene sequence of *Halobacterium* sp. GP5 1-1.**

CCATTGCTATCGGAGTCCGATTTAGCCATGCTAGTTGTGCGGGTTTAGACCCGCAGCGGAAAGCTCAGTAACACGTGGCCAAACTACCCTGTGGATGGGAACAATCTCGGGAAACTGAGGCTAATTCCCAATAACGCTCCACCCCTGGAACGGGCGGAGCTGGAAACGCTACGGCGCCACAGGATGTGGCTGCGGTCGATTAGGTAGACGGTGGGGTAACGGCCCACCGTGCCAATAATCGGTACGGGTTGTGAGAGCAAGAGCCCGGAGACGGAATCTGAGACAAGATTCCGGGCCCTACGGGGCGCAGCAGGCGCGAAACCTTTACACTGTACGCAAGTGCGATAAGGGGACTCCGAGTGTGAAGGCATAGAGCCTTCACTTTTGTACACCGTAAGGTGGTGTACGAATAAGGGCTGGGCAAGACCGGTGCCAGCCGCCGCGGTAATACCGGCAGCCCGAGTGATGGCCGATATTATTGGGCCTAAAGCGTCCGTAGCTGGCCGGACAAGTCCGTTGGGAAATCTGTTCGCTTAACGAGCAGGCGTCCAGCGGAAACTGTTCGGCTTGGGACCGGAAGACCTGAGGGGTACGTCCGGGGTAGGAGTGAAATCCTGTAATCCTGGACGGACCACCGGTGGCGAAAGCGCCTCAGGAGGACGGATCCGACAGTGAGGGACGAAAGCTAGGGTCTCGAACCGGATTAGATACCCGGGTAGTCCTAGCTGTAAACGATGCCCGCTAGGTGTGGCGCAGGCTACGAGCCTGCGCTGTGCCGTAGGGAAGCCGAGAAGCGGGCCGCCTGGGAAGTACGTCTG
