# Supplementary material for: Microscopic and spectroscopic bioassociation study of uranium(VI) with an archaeal Halobacterium isolate
Source: PLoS One. 2022 Jan 13;17(1):e0262275. doi: 10.1371/journal.pone.0262275 (PMC8757991; doi:10.1371/journal.pone.0262275)
Supplement: S1 Fig — Based on an alignment of 16S rRNA gene sequences (aligned with ClustalX-MEGA 6.06). GenBank accession numbers are shown in brackets. (DOCX) [file pone.0262275.s002.docx]

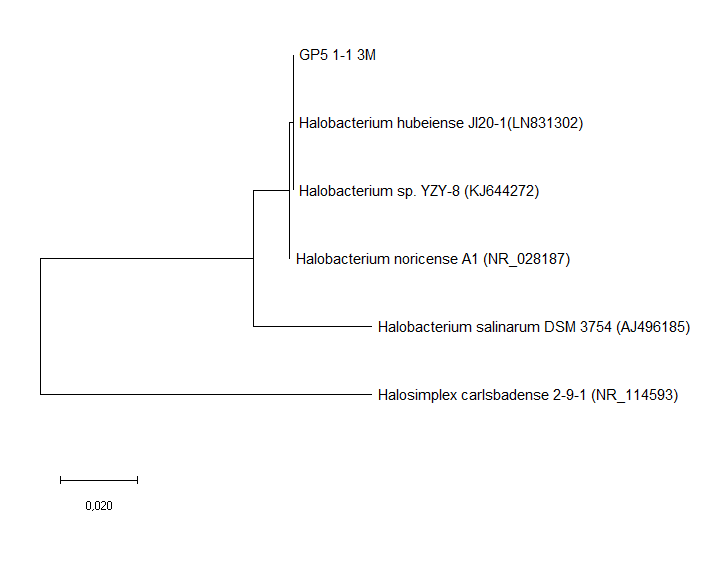


**S1 Fig. Phylogenetic dendrogram (neighbor-joining method) of Halobacterium sp. GP5 1-1 and its closest phylogenetic relatives.** Based on an alignment of 16S rRNA gene sequences (aligned with ClustalX-MEGA 6.06). GenBank accession numbers are shown in brackets
