# Supplementary material for: Microscopic and spectroscopic bioassociation study of uranium(VI) with an archaeal Halobacterium isolate
Source: PLoS One. 2022 Jan 13;17(1):e0262275. doi: 10.1371/journal.pone.0262275 (PMC8757991; doi:10.1371/journal.pone.0262275)
Supplement: S2 Fig — Based on the normalized luminescence intensities as a function of the incubation time at 30 μM uranium(VI) (brown = uranium(VI)-carboxylate, orange = uranium(VI)-phosphate). (DOCX) [file pone.0262275.s003.docx]

**S2 Fig.** **Species distribution in cell pellets of the uranium(VI) association experiment.** Based on the normalized luminescence intensities as a function of the incubation time at 30 µM uranium(VI) (brown = uranium(VI)-carboxylate, orange = uranium(VI)-phosphate).
