# Supplementary material for: Microscopic and spectroscopic bioassociation study of uranium(VI) with an archaeal Halobacterium isolate
Source: PLoS One. 2022 Jan 13;17(1):e0262275. doi: 10.1371/journal.pone.0262275 (PMC8757991; doi:10.1371/journal.pone.0262275)
Supplement: S3 Fig — (a) Spectrum 3 extracted using PARAFAC of the time-resolved emission spectra of the supernatants compared with the reference spectrum of lipopolysaccharide (LPS); (b) species distribution of the aquatic species as a function of the incubation time at 10 μM uranium(VI) under consideration of the bioassociation (red = free uranyl(VI), green = uranyl(VI)-carbonate complex, orange = uranyl(VI)-phosphate complex); in the sample after 48 h no uranium(VI) was still detectable. (DOCX) [file pone.0262275.s004.docx]

**S3 Fig.** **Extracted spectrum 3 and distribution of the aquatic uranium(VI) species at 10 µM uranium(VI).** (a) Spectrum 3 extracted using PARAFAC of the time-resolved emission spectra of the supernatants compared with the reference spectrum of lipopolysaccharide (LPS); (b) species distribution of the aquatic species as a function of the incubation time at 10 µM uranium(VI) under consideration of the bioassociation (red = free uranyl(VI), green = uranyl(VI)-carbonate complex, orange = uranyl(VI)-phosphate complex); in the sample after 48 h no uranium(VI) was still detectable.

In the bioassociation experiment with 10 µM uranium(VI), only spectra of the supernatants were recorded and evaluated. Besides the free uranyl(VI) and the carbonate complex, an aquatic phosphate species occurred in the supernatants, whose spectrum is shown in S4A Fig. The species distribution can be seen in S4B Fig.
