# Supplementary material for: Microscopic and spectroscopic bioassociation study of uranium(VI) with an archaeal Halobacterium isolate
Source: PLoS One. 2022 Jan 13;17(1):e0262275. doi: 10.1371/journal.pone.0262275 (PMC8757991; doi:10.1371/journal.pone.0262275)
Supplement: S4 Fig — (t = 24 h, DBM = 0.5 mg/mL, [NaCl] = 3 M). (DOCX) [file pone.0262275.s005.docx]

**S4 Fig.** **Concentration-dependent association of uranium(VI) onto H. sp. GP5 1-1**. (t = 24 h, DBM = 0.5 mg/mL, [NaCl] = 3 M).

Besides the investigations of the association kinetics with 10 and 30 µM uranium(VI), a concentration dependent experiment with uranium(VI) concentrations between 10 and 60 µM was performed. The incubation time for the samples was 24 h. S5 Fig shows a linear correlation between the amounts of bioassociated uranium(VI) and the initial concentration.
