# Supplementary material for: Microscopic and spectroscopic bioassociation study of uranium(VI) with an archaeal Halobacterium isolate
Source: PLoS One. 2022 Jan 13;17(1):e0262275. doi: 10.1371/journal.pone.0262275 (PMC8757991; doi:10.1371/journal.pone.0262275)
Supplement: S5 Fig — Cells were dyed with LIVE/DEAD® BacLight™ Bacterial Viability Kit; green = living cells, red = dead cells (after 24 h of incubation). (DOCX) [file pone.0262275.s006.docx]

**S5 Fig.** **Live/dead staining of the cells of *H.* sp. GP5 1-1 at different uranium(VI) concentrations.** Cells were dyed with LIVE/DEAD® BacLight™ Bacterial Viability Kit; green = living cells, red = dead cells (after 24 h of incubation).

The live dead images of the concentration-dependent experiment (S6 Fig) show an increasing size of the cell agglomerates and an increasing proportion of dead cells with increasing uranium(VI) concentrations.
