# Supplementary material for: Microscopic and spectroscopic bioassociation study of uranium(VI) with an archaeal Halobacterium isolate
Source: PLoS One. 2022 Jan 13;17(1):e0262275. doi: 10.1371/journal.pone.0262275 (PMC8757991; doi:10.1371/journal.pone.0262275)
Supplement: S6 Fig — Spectra extracted using PARAFAC of the time-resolved emission spectra compared with the reference spectra of (a) uranyl(VI)-hydrolysis complex, (b) uranyl(VI)-carbonate complex. (DOCX) [file pone.0262275.s007.docx]

**S6 Fig.** **Extracted spectra of the uranium(VI) species in the supernatants of the concentration dependent experiment.** Spectra extracted using PARAFAC of the time-resolved emission spectra compared with the reference spectra of (a) uranyl(VI)-hydrolysis complex, (b) uranyl(VI)-carbonate complex.
